# SIRT1-targeted miR-543 autophagy inhibition and epithelial–mesenchymal transition promotion in *Helicobacter pylori* CagA-associated gastric cancer

**DOI:** 10.1038/s41419-019-1859-8

**Published:** 2019-08-19

**Authors:** Yanyan Shi, Ziwei Yang, Ting Zhang, Lijuan Shen, Yuan Li, Shigang Ding

**Affiliations:** 1 Research Center of Clinical Epidemiology, Peking University Third Hospital, 100191 Beijing, PR China; 2 Department of Clinical Laboratory Medicine, The First Affiliated Hospital of China Medical University, Shenyang, 110001 Liaoning PR China; 3 Department of Microbiology, Peking University Health Science Center, 100191 Beijing, PR China; 4 Department of Gastroenterology, Affiliated Hospital of Qinghai University, 810001 Xining, PR China; 5 Department of Gastroenterology, Peking University Third Hospital, 100191 Beijing, PR China

**Keywords:** Cancer, Autophagy

## Abstract

Gastric cancer is an important cause of death worldwide with *Helicobacter pylori* (*H. pylori*) considered a leading and known risk factor for its development. More particularly and despite the underlying mechanisms not being very clear, studies have revealed that the *H. pylori* cytotoxin-associated gene A (CagA) protein plays a key role in this process. In this study it was found that *H. pylori* increased the expression of miR-543 in human gastric cancer tissue when compared with *H. pylori*-negative gastric cancer tissue samples. In vitro experiments showed that increased expression of miR-543 induced by CagA is a strong promoter of cell proliferation, migration, and invasion. Conversely, a miR-543 inhibitor suppressed or reversed these effects. It was furthermore found that silencing miR-543 inhibited autophagy and led to epithelial–mesenchymal transition (EMT) under in vitro. The mechanisms by which miR-543 targets SIRT1 to downregulate autophagy was also described. The results suggest that in the progression of *H. pylori*-associated gastric cancer, CagA induces overexpression of miR-543, which subsequently targets SIRT1 to suppress autophagy. This may be followed by increased expression of EMT causing cell migration and invasion. Consequently, miR-543 might be considered a therapeutic target for *H. pylori*-associated gastric cancer.

## Introduction

Gastric cancer, universally known as one of the most serious malignancies, is the fourth most commonly diagnosed cancer and the second most frequent cause of cancer-related deaths worldwide^1^. More than 1 million cases and 783,000 gastric cancer-associated deaths occurred worldwide in 2018^2^. *Helicobacter pylori* (*H. pylori*), a gram-negative pathogen, specifically colonizes the gastric epithelium^3^. This organism is present in approximately half of the global population^4^. A notable consequence of infection with *H. pylori* is an increased risk of atrophic gastritis and metaplastic gastritis. These can potentially progress to various diseases, such as peptic ulcers, gastric lymphoma or even gastric cancer^5,6^. Further studies have revealed that various virulence factors associated with *H. pylori* constitute a major cause of these diseases^7,8^. It has been reported that the cytotoxin-associated gene A (*CagA*), which is a virulence factor encoded by the cag pathogenicity island (cag-PAI) in 89% of *H. pylori* strains, plays critical roles in the pathogenicity of *H. pylori*^9,10^. However, the specific molecular mechanism(s) of *H. pylori*-associated gastric cancer is still unclear.

An increasing number of studies have suggested that microRNAs (miRNAs) play a key role in *H. pylori*-associated gastric diseases^11–13^. Approximately 19–25 nucleotides in length, miRNAs are short RNA transcripts that do not, or have a limited ability to, encode for proteins, and negatively regulate specific target gene expression^14–17^. In previous reports, miRNA microarrays have demonstrated that miR-543 expression is significantly increased in *H. pylori*-positive (HP+) gastric cancer tissue when compared with *H. pylori*-negative (HP−) cancer tissue samples^18^. It has also been demonstrated that miR-543 has the ability to accelerate cell proliferation, migration, and invasion in the development of various malignancies. These malignancies include colorectal, prostate, and lung cancer^19–21^.

Bioinformatics analysis has further predicted that miR-543 directly targets the 3ʹ-UTR of *SIRT*1 and leads to the repression of gene expression. Previous studies have shown that SIRT1-mediated deacetylation and activation of LKB1 results in increased phosphorylation of AMPK, which can trigger autophagy^22–24^. It has been revealed that autophagy is downregulated in human gastric mucosa with CagA-positive (CagA+) *H. pylori* infections and that the inhibition of autophagy increases cytokine production. This, in turn, promotes the *H. pylori*-induced inflammatory response^25^. When it comes to *H. pylori*-induced cancer, further reports found that downregulation of p14ARF, as induced by *H. pylori* CagA, leads to the inhibition of autophagy^26^. We therefore postulated that miR-543 expression is increased by CagA, targets *SIRT1*, and inhibits autophagy in *H. pylori*-associated gastric cancer.

In the present study, expression of miR-543 and SIRT1 in human gastric cancer and paracarcinoma tissue samples were investigated. We analyzed cell proliferation, migration, and invasion in gastric cancer cell lines after infection with CagA+ *H. pylori*. Epithelial–mesenchymal transition (EMT) and autophagy in cells with different expression levels of miR-543 and SIRT1 were also studied. Our findings indicated that miR-543 may be considered a therapeutic target for *H. pylori*-associated gastric cancer.

## Results

### *H. pylori* CagA increases expression of miR-543

**Fig. 1:**
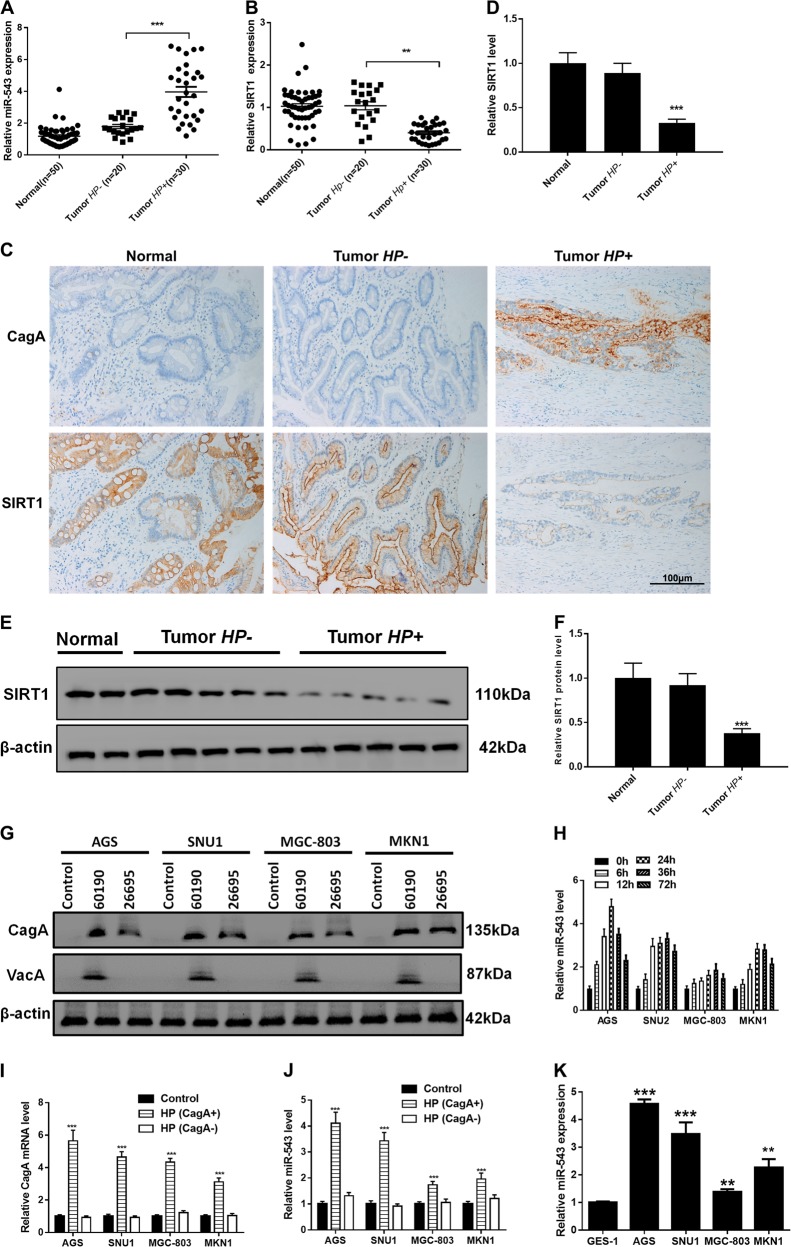
*H. pylori* CagA increased expression of miR-543. **a** RT-PCR results of miR-543 expression. **b** RT-PCR results of SIRT1 expression. **c** IHC experiments of CagA and SIRT1 in normal gastric tissue; HP+ or HP−. **d** Quantified IHC results. **e** Western blot results of SIRT1 in tumor tissues; HP+ or HP−. **f** Quantified western blot results. **g** Western blot results of CagA and VacA in AGS, SNU1, MGC-803, and MKN1; infected with strain 60190 or 26695. **h** RT-PCR results of miR-543 in AGS, SNU1, MGC-803, or MKN1 cell lines after infected with H. pylori CagA+ strain 26695 in different hours. **i** RT-PCR results of CagA in AGS, SNU1, MGC-803, or MKN1 cell lines after infected with H. pylori CagA+ or CagA− strain in 24 h. **j** RT-PCR results of miR-543 in AGS, SNU1, MGC-803, or MKN1 cell lines after infected with H. pylori CagA+ or CagA− strain in 24 h. **k** RT-PCR results of miR-543 in GES-1, AGS, SNU1, MGC-803, or MKN1 cells. Data are expressed as the mean ± SD (*n* = 3). **P < 0.01, ***P < 0.001 compared with control

The CagA protein has been considered a major cause of *H. pylori*-induced gastric carcinoma^27^. Most of the *H. pylori* hosts in China were infected with CagA+ strains^28,29^. We collected gastric cancer and paracarcinoma tissue samples from 50 patients and divided the samples into HP+ (all CagA+) and HP− groups. RT-PCR analysis demonstrated that, when compared with HP− tumor or normal tissue samples, the expression of miR-543 was significantly increased and SIRT1 expression decreased in HP+ tumor tissue (Fig. 1a, b). Immunohistochemistry (IHC) results confirmed the downregulated expression of SIRT1 and upregulated expression of CagA+ in HP+ tissue (Fig. 1c, d). Western blot analyses confirmed the overexpression of SIRT1 in HP+ tissues (Fig. 1e, f). Consequently, the SNU1, AGS, MGC-803, and MKN1 gastric cancer cell lines were infected with *H. pylori* strain 26695 or strain 60190 (both CagA+). Following 24 h of incubation, western blot analyses showed that *H. pylori* strain 26695 is VacA-negative (Fig. 1g). As shown in Fig.1h, AGS, SNU1, MGC-803, or MKN1 cell lines after infected with H. pylori CagA+ strain 26695 in different hours. In most of cell lines, the expression of miR-543 in 24 h was highest. So we used 24 h for further experiment. RT-PCR results showed that expression levels of both CagA and miR-543 were increased in each cell line after infection with CagA+ *H. pylori* (strain 26695), especially in AGS and SNU1 cells (Fig. 1h–j). When compared with normal gastric mucosa cells (GES-1), RT-PCR data showed that expression of miR-543 was increased in CagA + *H. pylori* AGS, SNU1, MGC-803, and MKN1 cells.

### The overexpression of miR-543 promotes the accelerating effect of CagA+ *H. pylori* on cell proliferation

**Fig. 2:**
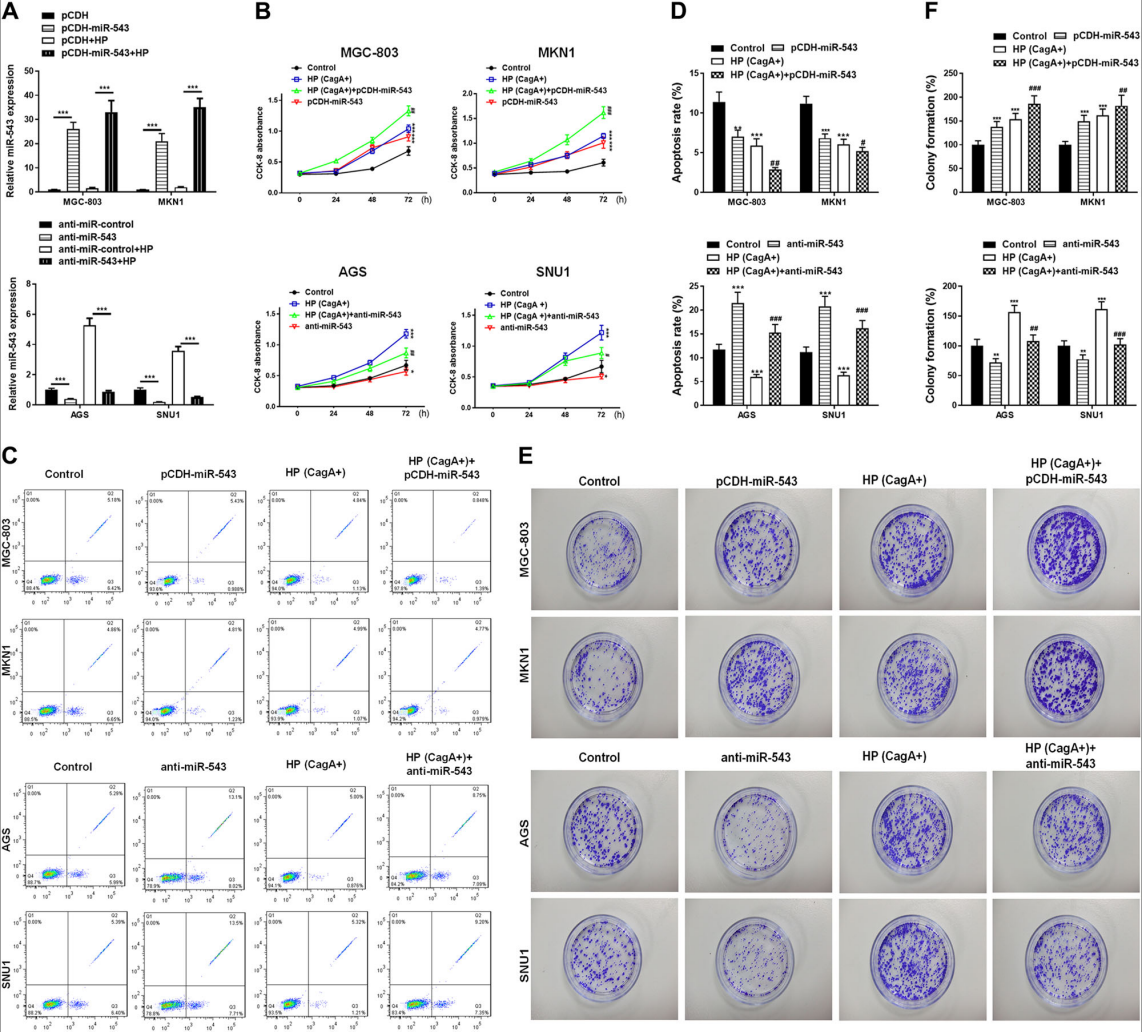
miR-543 overexpression promoted the accelerating effect of CagA+ *H. pylori* on cell proliferation. **a** RT-PCR results of miR-543 expression in MGC-803 and MKN1 cells transfected with pCDH-miR-543 vectors or AGS and SNU1 transfected with the anti-miR-543 vector; infected with HP or not. **b** CCK-8 cell proliferation analyses of MGC-803 (HP (CagA+) or pCDH-miR-543 or both HP (CagA+) and pCDH-miR-543), MKN1 (HP (CagA+) or pCDH-miR-543 or both HP (CagA+ ) and pCDH-miR-543), AGS (HP (CagA+) or anti-miR-543 or both HP (CagA+) and anti-miR-543), and SNU1 (HP (CagA+) or anti-miR-543 or both HP (CagA+) and anti-miR-543). **c** AV-FITC staining apoptosis results of four kinds of cell lines processed as shown in Fig. 2c. **d** Quantitative analysis of apoptosis. **e** Colony formation cell proliferation analyses results. **f** Quantitative analysis of cell proliferation. Data are expressed as the mean ± SD (*n* = 3). **P < 0.01, ***P < 0.001 versus control group; ^##^*P* < 0.01, ^###^*P* < 0.001 versus CagA+ HP group

The miR-543 overexpression vector pCDH-miR-543 was constructed and transfected into MGC-803 and MKN1 cells (with or without *H. pylori* infected). Similarly, an anti-miR-543 vector, which is an inhibitor of miR-543, was constructed and transfected into SNU1 and AGS cells (with or without *H. pylori* infected). RT-PCR analysis showed that the expression of miR-543 was increased in MGC-803 and MKN1 cells, and decreased in SNU1 and AGS cells (Fig. 2a). Cell proliferation was determined by CCK-8 analysis after the cells were infected with *H. pylori* (CagA+) and transfected with mimics. Data showed that infection with *H. pylori* (CagA+) promoted cell proliferation in all four cell lines. Overexpression of miR-543 increased the promotion of cell proliferation, while anti-miR-543 inhibited proliferation (Fig. 2b). Colony formation assays showed similar results (Fig. 2e, f). The apoptosis rate was assessed by flow cytometry with Annexin V (AV)-fluorescein isothiocyanate (FITC) staining. Results showed that *H. pylori* (CagA+) blocked apoptosis and that pCDH-miR-543 enhanced this trend (Fig. 2c). Conversely, anti-miR-543 eliminated the inhibitory effect that CagA had on apoptosis (Fig. 2c). Transfection with pCDH-miR-543 alone can also promote cell proliferation and inhibit apoptosis, while anti-miR-543 had an opposite effect (Fig. 2b–e). Therefore, AV/FITC apoptosis detection verified the aforementioned conclusions (see above).

### Cell migration, invasion, and EMT is increased by miR-543 overexpression in CagA+ *H. pylori* infected cells

**Fig. 3:**
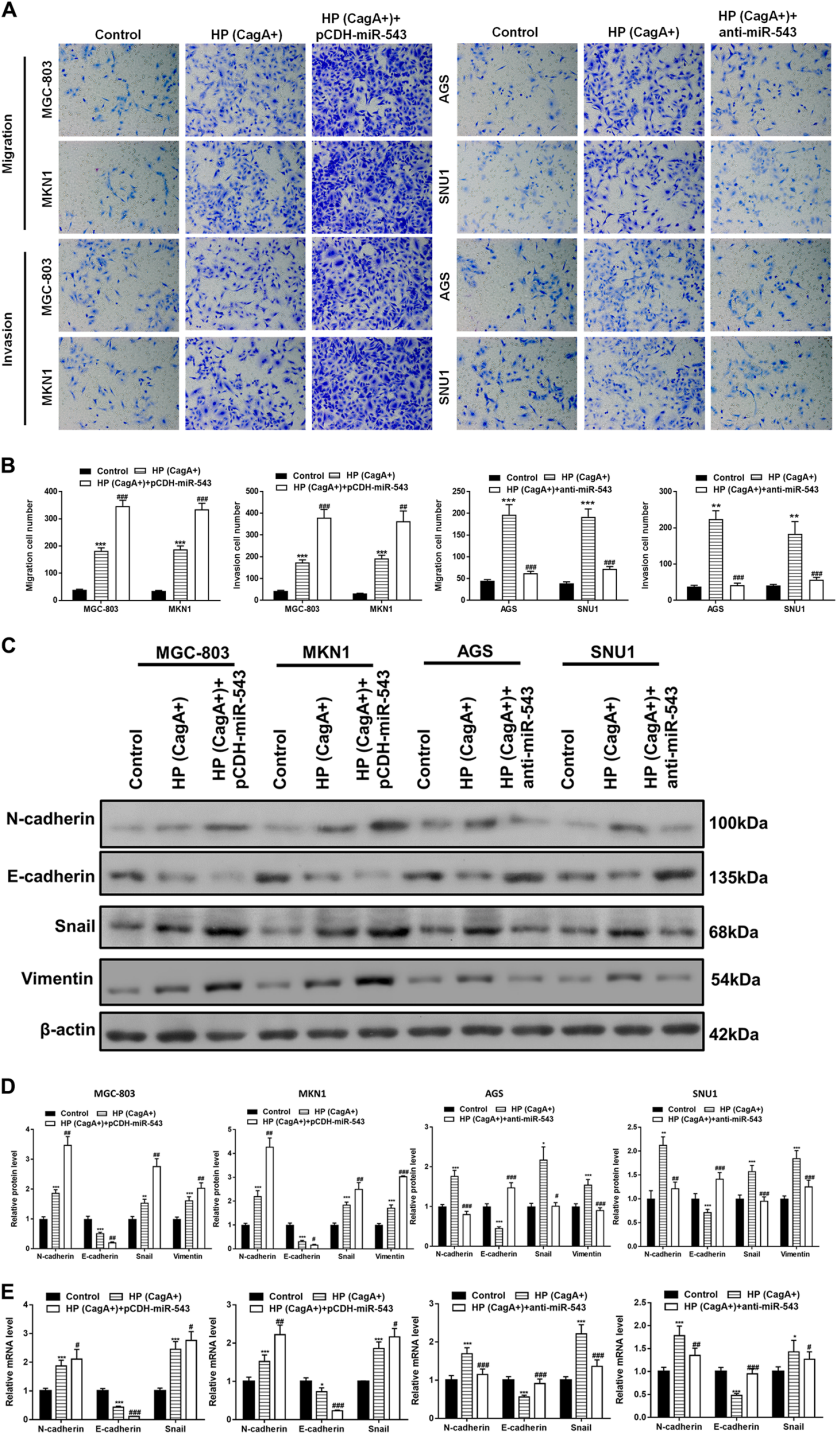
Promotion of cell migration, invasion, and EMT was increased by miR-543 overexpression in CagA + *H. pylori* cells. **a** Transwell chamber results of MGC-803 (HP (CagA+) or both HP (CagA+) and pCDH-miR-543), MKN1 (HP (CagA+) or both HP (CagA+) and pCDH-miR-543), AGS (HP (CagA+) or both HP (CagA+) and anti-miR-543), and SNU1 (HP (CagA+) or both HP (CagA+) and anti-miR-543). **b** Quantitative analysis of cell migration and invasion of four kinds of cell lines processed as shown in Fig. 3a. **c** Western blot results of EMT-related N-cadherin, E-cadherin, Snail, and Vimentin proteins. **d** Quantified western blot results and RT-PCR results of EMT-related genes. Data are expressed as the mean ± SD (*n* = 3). *P < 0.05, **P < 0.01, ***P < 0.001 versus control group; ^#^*P* < 0.05, ^##^*P* < 0.01, ^###^*P* < 0.001 versus CagA+HP group

The migration and invasion abilities of gastric cancer cells were assessed using Transwell assays. The results using MGC-803 and MKN1 cells showed that infection with the CagA+ *H. pylori* strain promoted cell migration and invasion. Cell migration and invasion effects were further promoted with the overexpression of miR-543 (Fig. 3a, b). Similarly, although CagA+ *H. pylori* infection promoted migration and invasion of AGS and SNU1 cells, anti-miR-543 transfection reversed these effects (Fig. 3a, b). To further confirm that miR-543 increased cell migration and invasion through the promotion of EMT, the expression of EMT-related proteins N-cadherin, E-cadherin, Snail, and Vimentin were examined by western blots and RT-PCR. Following infection with CagA+ *H. pylori*, the results showed that expression of N-cadherin and Snail were increased at both the protein and transcription levels. This trend was heightened by miR-543 overexpression and suppressed by anti-miR-543 (Fig. 3c–e). Similarly, the inhibitory effect of CagA+ *H. pylori* on E-cadherin expression was enhanced by pCDH-miR-543, but suppressed by anti-miR-543 (Fig. 3c–e). These results suggested that miR-543 had the ability to upregulate the EMT, and to promote cell migration and invasion.

### SIRT1 is a direct target of miR-543

**Fig. 4:**
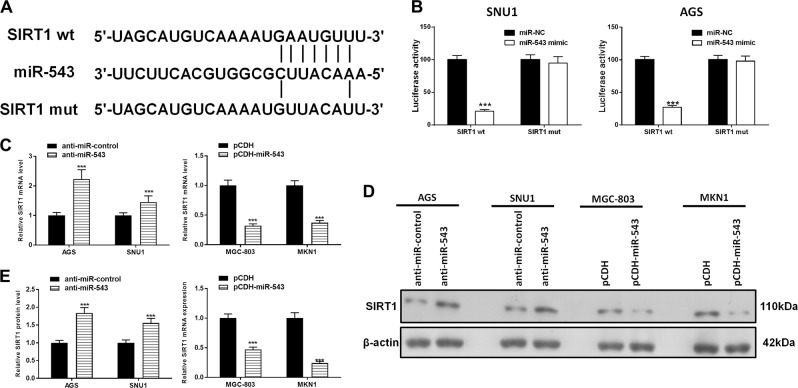
SIRT1 is a direct target of miR-543. **a** Complementary sequences between miR-543 and the 3ʹ-UTR of SIRT1 or a mutated version of SIRT1 were obtained using publicly available algorithms. **b** Dual-luciferase reporter assays of SNU1 and AGS transfected by a mimic of miR-543 (with SIRT1-WT or SIRT1-MUT). **c**, **d** Western blot results of SIRT1 and quantified. **e** RT-PCR detection of SIRT1 expression in MGC-803 and MKN1 cells transfected with pCDH-miR-543, or AGS, and SNU1 cells transfected with anti-miR-543. Data are expressed as the mean ± SD (*n* = 3). ***P < 0.001 versus control group

Bioinformatics analyses (http://www.targetscan.org) predicted that SIRT1 was a potential functional target of miR-543 (Fig. 4a). Dual-luciferase reporter assays showed that overexpression of miR-543 reduced the intensity of fluorescence in SNU1 and AGS cells transfected with SIRT1 wild-type (WT) vectors but had no effect on the cells that were transfected with SIRT1-mutant (MUT) vectors (Fig. 4b). Subsequent analyses confirmed that miR-543 significantly inhibited SIRT1 expression at both the transcript and protein levels in gastric cancer cells (Fig. 4c–e). Collectively, the results suggested that miR-543 directly targeted SIRT1 and caused translational repression in gastric cancer cells.

### SIRT1 is targeted by miR-543 to suppress autophagy in gastric cancer cells

**Fig. 5:**
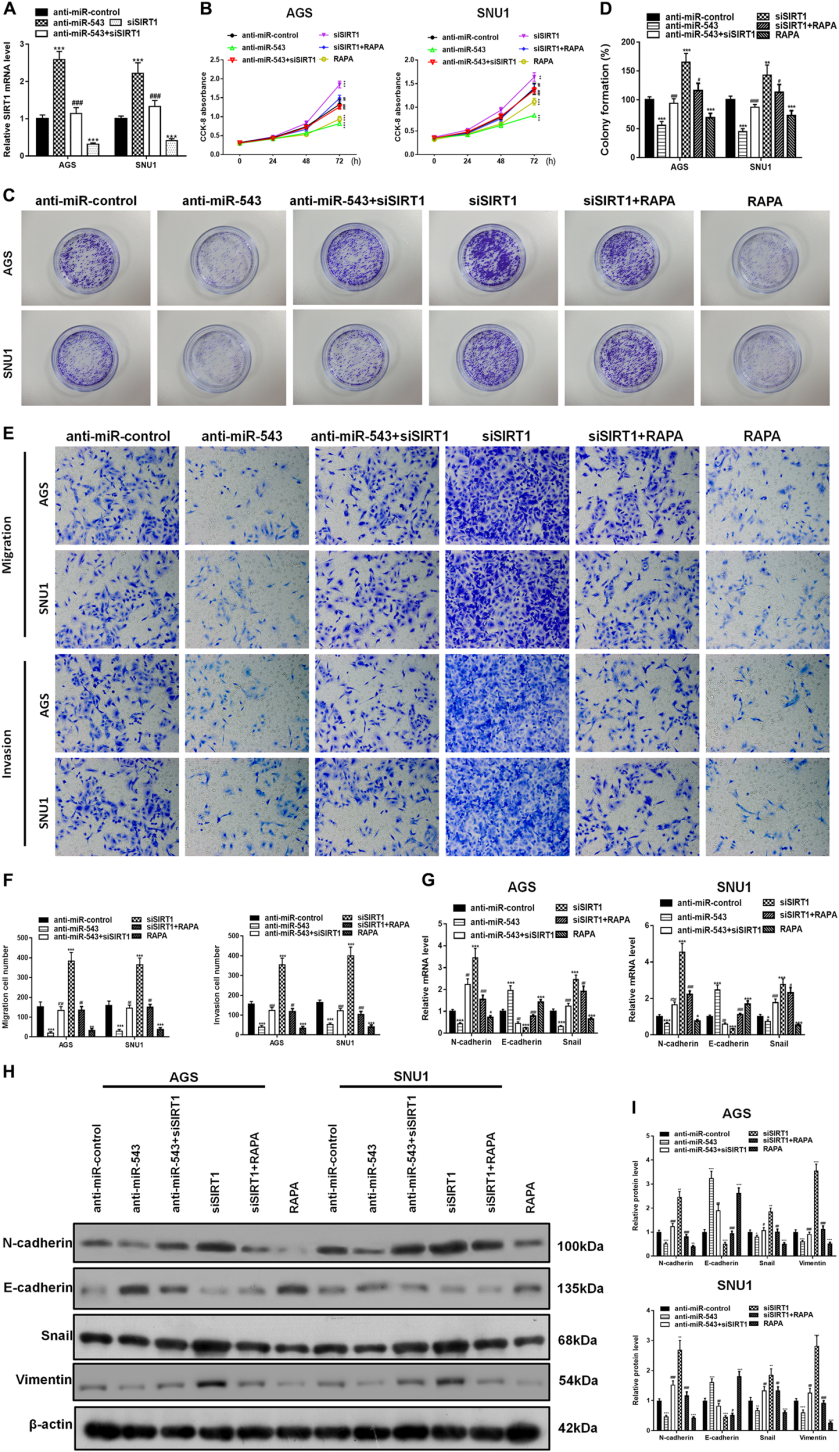
SIRT1 was targeted by miR-543 to suppress cell proliferation, migration and invasion. **a** RT-PCR analyses of SIRT1 in AGS and SNU1 cells transfected with anti-miR-543, siSIRT1, or both anti-miR-543 and siSIRT1. **b** CCK-8 cell proliferation analyses of AGS and SNU1 cells transfected with anti-miR-543, siSIRT1, or both anti-miR-543 and siSIRT1. Autophagy enhancer RAPA addition and siSIRT1 transfection results were also studied and compared. **c** Colony formation assays show the results of cell proliferation. **d** Quantitative analysis of colony formation. **e** Transwell chamber experiments on cell migration and invasion. **f** Quantitative results of migration and invasion. **g** RT-PCR detection of N-cadherin, E-cadherin, and Snail proteins in AGS and SNU1 cells. **h**, **i** Western blot detection of N-cadherin, E-cadherin, Vimentin, and Snail in AGS and SNU1 cells and quantified. Data are expressed as the mean ± SD (*n* = 3). *P < 0.05, **P < 0.01, ***P < 0.001 versus control group; ^#^*P* < 0.05, ^##^*P* < 0.01, ^###^*P* < 0.001 versus siSIRT1 group

SIRT1-silencing vectors (siSIRT1) were constructed and transfected into AGS and SNU1 cells. While RT-PCR analysis showed that transfection with anti-miR-543 increased the expression of SIRT1 (Fig. 3c), siSIRT1 effectively suppressed the increased expression of SIRT1 (Fig. 5a). Cell proliferation was determined by CCK-8 and colony formation assays after the cells had been infected with *H. pylori* (CagA+) and transfected with different mimics. The results indicated that, while both the anti-miR-543 vector and autophagy enhancer rapamycin (RAPA) inhibited proliferation of AGS and SNU1 cells, siSIRT1 reversed the inhibition of AGS and SNU1 cell proliferation (Fig. 5b). Colony formation assays mirrored these results (Fig. 5c, d). Analyses using transwell assays revealed that siSIRT1 mimics had the ability to reverse the inhibition of cell migration and invasion caused by anti-miR-543 or RAPA (Fig. 5e, f). Western blot analyses of EMT-related N-cadherin, E-cadherin, Snail, and Vimentin proteins indicated that both transfection of anti-miR-543 and addition of RAPA suppressed the EMT caused by *H. pylori* CagA. Western blot analyses also showed that siSIRT1 reversed the inhibitory effect (Fig. 5g–i).

**Fig. 6:**
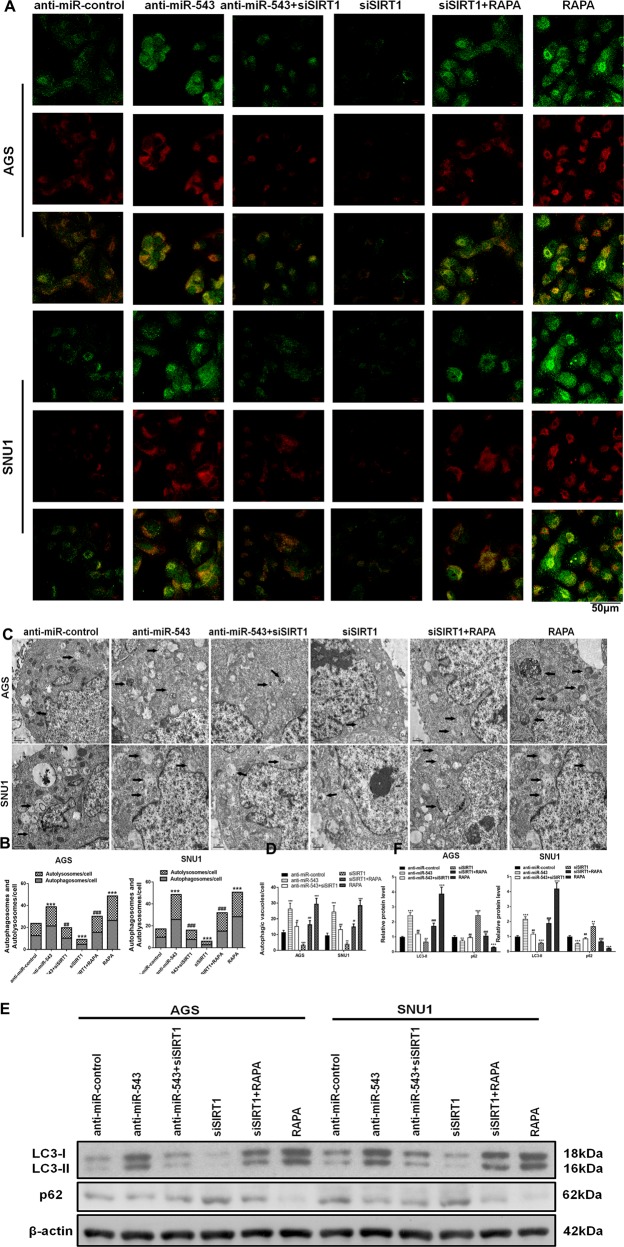
SIRT1 was targeted by miR-543 to suppress autophagy. **a** Detection of autophagy using mRFP-GFP-LC3 in AGS and SNU1 cells transfected with anti-miR-543, siSIRT1, or both anti-miR-543 and siSIRT1. Green puncta indicate the autophagosomes, while the red puncta represent the autolysosomes. Autophagy enhancer RAPA addition and siSIRT1 transfection results were also studied and compared. **b** Quantitative analysis of mRFP-GFP-LC3 detection. **c** The autophagic vacuoles (autophagosomes) were detected by transmission electron microscopy (TEM). The representative TEM images are shown and the typical autophagosomes are marked with arrows. **d** Quantitative analysis of autophagy detection by TEM. **e**, **f** Western blot detection of autophagy-related proteins LC3-I, LC3-II, and p62 and Quantified. Data are expressed as the mean ± SD (*n* = 3). *P < 0.05, **P < 0.01, ***P < 0.001 versus control group; ^#^*P* < 0.05, ^##^*P* < 0.01, ^###^*P* < 0.001 versus siSIRT1 group

In detecting and quantifying autophagy, mRFP-GFP-LC3 constructs were used. The results showed that both transfection of anti-miR-543 and addition of RAPA suppressed the inhibition of autophagy caused by *H. pylori* CagA. We use mRFP-GFP-LC3 adenovirus to monitor autophagic flux. The green puncta indicate the autophagosomes, while the red puncta represent the autolysosomes (Fig. 6a, b). And the results indicated that siSIRT1 downregulated the expression of autophagy to levels similar to those observed in the control group. Western blot analyses of autophagy-related proteins LC3 and p62, and transmission electron microscope (TEM) results were similar to the results obtained from the mRFP-GFP-LC3 experiments (Fig. 6c–f). Collectively, these results suggested that miR-543 targeted SIRT1, thus inhibiting CagA-mediated autophagy and EMT. Downregulation of autophagy and elevated levels of EMT significantly increased the rates of cell proliferation, migration, and invasion.

### SIRT1 is targeted by miR-543 to suppress autophagy in vivo

**Fig. 7:**
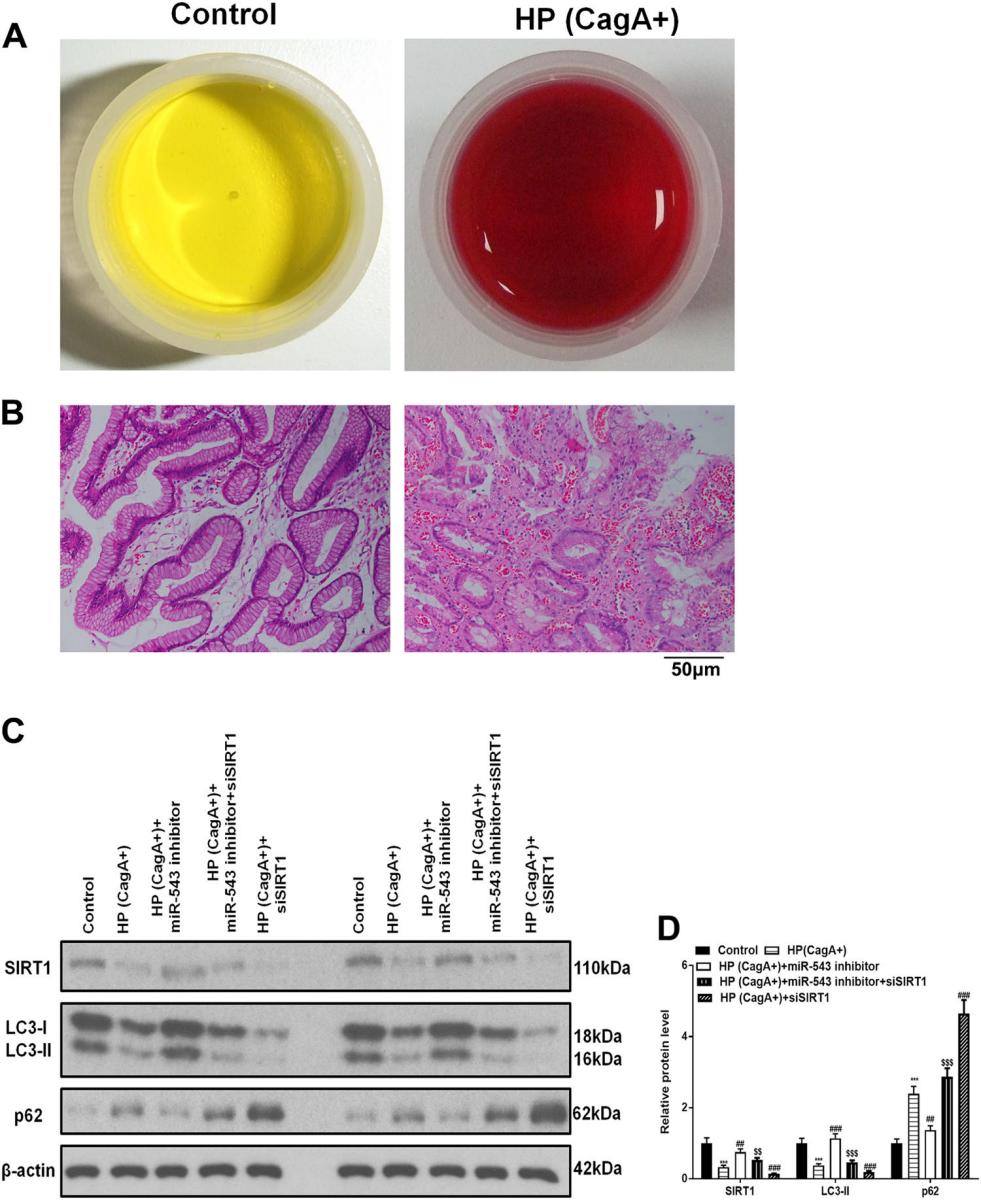
SIRT1 was targeted by miR-543 to suppress autophagy in vivo. **a** Rapid urease test of *H. pylori* infection on C57BL/6 mice. **b** Hematoxylin-eosin staining image of control and HP (CagA+) group in mice gastric epithelium. **c**, **d** Western blot detection of SIRT1 and autophagy-related proteins LC3-I, LC3-II, and p62 and quantified. Data are expressed as the mean ± SD (n = 8). **P < 0.01, ***P < 0.001 versus control group; ^#^*P* < 0.05, ^##^*P* < 0.01, ^###^*P* < 0.001 versus HP(CagA+) group, ^$$^*P* < 0.01, ^$$$^*P* < 0.001 versus HP(CagA+) + miR-543 inhibitor group

To determine if the above results could be recapitulated in vivo, C57BL/6 mice were inoculated with *H. pylori* strain 26695 (CagA+), anti-miR-543, and siSIRT1. Gastric epithelium tissue samples were then obtained and analyzed. Rapid urease tests showed that *H. pylori* had infected the samples (Fig. 7a). Hematoxylin-eosin staining confirmed the infection (Fig. 7b). Consequently, western blot analyses showed that the expressions of LC3-I and LC-II were decreased by *H. pylori* CagA. Western blot analyses also showed that the miR-543 inhibitor reversed the reduction effect of miR-543 on LC3, while siSIRT1 suppressed the reversal effect of anti-miR-543 (Fig. 7c, d). In contrast to the results observed for LC3-I and LC-II, an increased expression of p62, a protein that negatively regulates autophagy, was observed (Fig. 7c, d). However, these results validated the hypothesis that miR-543 targets SIRT1 thereby inhibiting CagA-mediated autophagy in vivo.

## Discussion

*H. pylori*-induced chronic gastritis is the leading known risk factor of gastric cancer^30^. The pathogenicity of *H. pylori* is largely due to its virulent components, such as flagella, lipopolysaccharides, VacA, and cytotoxin-related gene pathogenic island (cagPAI). CagPAI is one of the most important and widely studied virulence components. The cagPAI gene of *H. pylori* encodes virulence factors CagA and T4SS. CagA protein can be injected into gastric epithelial cells through T4SS and plays an important role in *H.* *pylori*-induced inflammation and tumorigenesis^31^. An increasing number of studies have suggested that miRNAs play a key role in gastric diseases that are induced by CagA+ strains of *H. pylori*^19–21,31^. As revealed by miRNA microarray analyses, miR-543 expression is significantly increased in HP+ gastric cancer tissue when compared with HP− cancer tissue^18^. The purpose of this study was therefore to determine the role that miR-543 plays in *H. pylori*-induced gastric cancer and also to assess its specific molecular mechanism.

In this study it was confirmed that *H. pylori* CagA increased the expression of miR-543 in human gastric cancer tissue and four different gastric cancer cell lines (SNU1, AGS, MGC-803, and MKN1). To investigate whether the expression of miR-543 was responsible for the pathogenicity of *H. pylori* CagA, we constructed and transfected gastric cancer cells with a miR-543 overexpression vector (pCDH-miR-543) or miR-543 inhibitor (anti-miR-543). Our in vitro experiments indicated that miR-543 overexpression increased cell proliferation induced by *H. pylori* CagA. In addition, the examination of EMT-related N-cadherin, E-cadherin, and Snail proteins showed that *H. pylori* CagA increased the EMT, thereby causing an increase in cell migration and invasion. Lastly, it was found that the effects of CagA were enhanced by miR-543 overexpression.

To further investigate the molecular mechanism of miR-543, bioinformatics analyses predicted that SIRT1 served as a potential functional target. Dual-luciferase reporter assays revealed that miR-543 directly targeted SIRT1 and caused translational repression in gastric cancer cells. SIRT1 has been associated with autophagy through the activation of AMPK^22–24^. Conversely, *H. pylori* CagA has been shown to downregulate autophagy^26^. Further reports have revealed that autophagy can suppress EMT under several circumstances^32–34^. To identify whether miR-543 targets SIRT1 to inhibit autophagy and facilitate EMT, siSIRT1 mimics were constructed and cells transfected. CCK-8, colony formation, and western blot assays collectively revealed that siSIRT1 reversed the inhibiting effect of anti-miR-543 or RAPA on EMT-induced cell migration and invasion. Autophagy detection by mRFP-GFP-LC3, TEM, and western blot analyses of autophagy-related proteins confirmed that siSIRT1 downregulated autophagy. Follow-up in vivo experiments supported these results.

Collectively, this study showed that in the process of *H. pylori* CagA-inducing gastric cancer, miR-543 promoted cell proliferation. We also found that, when induced by *H. pylori* CagA, miR-543 overexpression targeted SIRT1, thereby leading to the inhibition of autophagy. The inhibition of autophagy promoted EMT, which led directly to cell migration and invasion. These results provide new insights into the molecular mechanism(s) of gastric cancer, with miR-543 serving as a potential therapeutic target for gastric cancer linked to *H. pylori*.

## Materials and methods

### Clinical specimens and cell lines

The current study included 50 gastric cancer patients who attended Peking University Third Hospital, China from 2015 to 2017. In total, 20 HP−, 30 HP+, and 50 paracarcinoma tissue samples were collected via biopsy or surgical resection. Samples were divided and either frozen in liquid nitrogen and stored at −80 °C or preserved in RNAlater (Ambion, Austin, TX, USA) at −20 °C. The study was conducted in accordance with the Declaration of Helsinki and approved by Peking University Third Hospital. Informed consent was obtained for study participation from all patients or their direct relatives.

The human gastric cancer cell lines SNU1, AGS, MGC-803, and MKN1 were obtained from the Shanghai Institute of Cell Biology (Shanghai, China). Cell lines were maintained in Dulbecco’s Modified Eagle’s medium (DMEM, Hyclone, UT, USA) supplemented with 10% fetal bovine serum (FBS, Hyclone, UT, USA), 1% penicillin–streptomycin amphotericin B solution, 1% l-glutamate, and 1% nonessential amino acids in a 37 °C incubator containing 5% CO_2_. With the exception of the FBS, all remaining reagents were obtained from Gibco (Gaithersburg, MD, USA) and Biological Industries (Beit Haemek, Israel).

### *H. pylori* strains and cell coculture

*H. pylori* bacterial strains 60190 (ATCC 49503, CagA+/VacA+), 26695 (ATCC 700392, CagA+/VacA−), and Tx30a (ATCC 51932, CagA−) were obtained from the American Type Culture Collection (Manassas, VA, USA) and then treated. Bacteria were cultured on 5% horse blood agar plates (Oxoid Ltd, Basingstoke, UK) in humidified incubators with 5% CO_2_ at 37 °C. The *H. pylori* strains (both CagA+ and CagA−) were inoculated into Brucella broth containing 5% FBS under microaerophilic conditions (5% O2, 10% CO2 and 85% N2) at 37 °C. For *H. pylori* infection, SNU1, AGS, MGC-803, and MKN1 cells were seeded into six-well plates with antibiotic-free cell culture medium and cultured to a confluency of 80–90%. The *H. pylori* bacterium was harvested, resuspended with phosphate-buffered saline (PBS) and added to the gastric cancer cells at a multiplicity of infection ratio of 100:1 as previously described^35^. The *H. pylori*-infected gastric cancer cells were incubated for either 6, 12, 24, 36, or 72 h, and then isolated.

### Immunohistochemistry (IHC)

Thawed samples were fixed in 4% formalin and embedded in paraffin for histopathological analyses. Samples were deparaffinized with xylol and then sliced into 4-µm sections. Sections were rehydrated using a graded ethanol series. A heat-induced epitope protocol was used for antigen-retrieval (95 °C for 40 min). Samples were incubated in methanol containing 0.3% hydrogen peroxide to block endogenous peroxidase. Samples were blocked with serum (Vectastain Elite ABC kit; Vector Laboratories Inc., Burlingame, CA, USA) and then incubated overnight at 4 °C with polyclonal rabbit anti-human SIRT1 antibody at a concentration of 1:1000 (Abcam, Shanghai, China). After washing three times in TBST (150 mM NaCl, 10 mM Tris-HCl, pH 7.6), sections were incubated with a secondary antibody for 20 min at room temperature. Peroxidase-conjugated biotin-streptavidin complex (Dako, Glostrup, Denmark) was then applied to the sections for 20 min. Sections were visualized with 3, 3ʹ-diaminobenzidine and counterstained with hematoxylin. The negative control used nonimmune serum instead of primary antibody.

### Plasmid construction

The human miR-543 precursor and human SIRT1 coding sequence were cloned into the mammalian expression vector pcDNA3.1(+) (Invitrogen, Shanghai, China) at the KpnI and XhoI cut sites (TaKaRa, Dalian, China) to generate stably-transfected gastric cancer cell lines. Virus was produced in 293 T cells cotransfected with the pCDH lentiviral vector and associated packaging plasmid.

### Cell transfection

Oligonucleotides for a miR-543 mimic and an inhibitor of miR-543 (anti-miR-543), were used (Thermo Scientific, Lafayette, CO, USA) for overexpression or inhibition of miR-543, respectively.

### Protein extraction and western blotting

Proteins were extracted from subconfluent cell cultures and subjected to western blot analyses. After blocking with 5% nonfat milk in PBS-T for 1 h at room temperature, the membranes were blotted with primary antibody, followed by blocking, and incubation with a peroxidase-conjugated secondary antibody. Bound antibodies were visualized using enhanced chemiluminescence (Bio-Rad, Hercules, CA, USA). The primary antibodies against SIRT1, Snail, N-cadherin, E-cadherin, p62, LC3, and β-actin were purchased from Cell Signaling Technology (Danvers, MA, USA). Signals were detected using a FluorChem E system (Alpha Innotech Corp, Santa Clara, CA, USA).

### RNA extraction and real-time PCR

Total RNA was extracted using TRIzol (Life Technologies) as per the manufacturer’s protocol. RNA was reverse-transcribed into cDNA with the Super-Script First-Strand cDNA System (Invitrogen, Carlsbad, CA, USA), and amplified with the Platinum SYBR Green qPCR SuperMix-UDG (Invitrogen). We prepared a master mix for qPCR testing using Platinum SYBR Green qPCR SuperMix-UDG, forward and reverse primers, and template cDNA (10 ng). The reaction conditions were 95 °C for 5 min, and then 32 cycles at 95 °C for 15 s and 1 min at 60 °C and 30 s at 72 °C. Beta-actin was used as an internal control. The relative expression levels of mRNA were quantified using the ^ΔΔ^Ct method.

### Cell proliferation assays

Cell proliferation was determined using CCK-8 assays (Dojindo Laboratories, Kumamoto, Japan). Briefly, gastric cancer cells were seeded into 96-well plates at an initial density of 5000 cells per well. Cells transfected with anti-miR-543 were plated on day 1. Ten microliters of the kit reagent were added to each well at 0, 24, 48, or 72 h after seeding. All plates were scanned using a microplate reader (Thermo Scientific) after a further 2 h. Cell proliferation was evaluated by absorbance at 450 nm.

### Apoptosis assay

Apoptosis was measured using an AV/FITC apoptosis detection kit (Bender Medsystem, Vienna, Austria). Briefly, cells cultured in 6-cm dishes were trypsinized, washed, and stained with FITC-conjugated anti-AV antibody in darkness for 15 min at room temperature, and then analyzed with a flow cytometer (FACSCalibur; Becton Dickinson, Franklin Lakes, NJ, USA).

### Colony formation assay

Cell proliferation was assessed using a soft agar colony formation assay. A six-well plate containing a 1.5 mL bottom layer and 0.5 mL top layer of agar was used (5.1 mg/mL; Difco Laboratories, Detroit, MI, USA). In total, 104 cells were transferred onto the bottom layer of each well, overlaid with a top layer of agar, and cultured at 37 °C in 5% CO_2_. Giemsa staining was used to quantify the formation of colonies on the 7th day.

### Cell migration and invasion assays

The migration and invasion abilities of gastric cancer cells were assessed using Transwell assays (Millipore, Billerica, MA, USA). GCs were seeded into uncoated or Matrigel-coated (BD Bioscience, Bedford, MA, USA) plates with 8-μm diameter pores in order to perform migration and invasion assays, respectively. The top chambers were seeded with cells at a density of 2 × 10^4^ cells/well in serum-free medium, while FBS with 10% serum was added to the lower chamber. After 24 h of incubation, nonmigratory cells found on the top surface of the filter were removed by rubbing with a cotton swab. Cells that had migrated to the lower chamber were quantified in five random fields using an optical inverted microscope at ×200 magnification (Nikon, Tokyo, Japan).

### Luciferase reporter assays

Luciferase reporter assays were conducted using the Dual-Luciferase Reporter Assay System (psiCHECK-2 vector; Promega, Madison, WI, USA). A fragment of the SIRT1 3ʹ-UTR containing either the predicted binding site for miR-543 or a mutated 3ʹ-UTR was inserted into the psiCHECK-2 vector. After verification by Sanger sequencing and using the Lipofectamine RNAiMAX kit (Invitrogen) according to the manufacturer’s instructions, the psiCHECK-2 vectors containing either the WT, or mutated SIRT1 3ʹ-UTR were transfected into gastric cancer cells. Gastric cancer cell lines presented either with or without a synthetic miR-543 mimic. At 36 h after transfection, luciferase activity was detected using a dual-luciferase reporter assay system and normalized against Renilla activity. Data were normalized to the luciferase activity of cells transfected with miR-control elements.

### Autophagic flux analysis

The mRFP-GFP-LC3 gastric cells were fixed with 4% paraformaldehyde and stained with 10 μM Hoechst 33342. Cell images were obtained from the Operetta High Content Imaging System (Perkin-Elmer, Waltham, MA, USA) and analyzed using Harmony Analysis Software (Perkin-Elmer). Cells were detected using green fluorescent protein (GFP) or red (mRFP) fluorescence. In merged images, autophagosomes stained puncta yellow, while autolysosomes stained puncta red. Autophagic flux was determined by the increased percentage of red only puncta in the merged images.

### Electron microscopy

Cells were fixed with 2.5% glutaraldehyde in phosphate buffer and stored at 4 °C until embedded. Cells were postfixed with 1% osmium tetroxide followed by an increasing gradient dehydration step using ethanol and acetone. Cells were then embedded in Araldite, following which ultrathin (50–60 nm) tissue sections were obtained, placed on uncoated copper grids, and stained with 3% lead citrate–uranyl acetate. Images were examined with a CM-120 electron microscope (Philips Microscopy, Hillsboro, OR, USA).

### *H. pylori* infection in vivo

Six to eight-week-old C57BL/6 mice (Institute of Zoology, Chinese Academy of Sciences, Shanghai, China) were inoculated orally with broth-cultured *H. pylori* strain 26695 (CagA+) at a concentration of 1 × 10^8^ CFU. Cultures were grown in 0.15 mL broth. Control mice were given PBS. A lentivirus expressing SIRT1 and miR-543 was purchased from HanBio. One hundred microliters of filter-purified lentivirus cocktail (1 × 10^5^IU/μl) or PBS was administered by intravenous injection weekly. Mice were killed 4 months after inoculation and gastric tissue collected for western blot analyses.

### Statistical analysis

The results are expressed as the mean ± SD. Statistical significance was evaluated by ANOVA, followed by Tukey–Kramer multiple comparison tests and by Student’s *t*-tests. A *p* < 0.05 denotes statistical significance.
